# Safety Analysis of Korean Cottage Industries’ *Doenjang*, a Traditional Fermented Soybean Product: A Special Reference to Biogenic Amines

**DOI:** 10.3390/foods12224084

**Published:** 2023-11-10

**Authors:** Ashutosh Bahuguna, Vishal Kumar, Gajanan Bodkhe, Srinivasan Ramalingam, SeMi Lim, Ah-ryeong Joe, Jong Suk Lee, So-Young Kim, Myunghee Kim

**Affiliations:** 1 Department of Food Science and Technology, Yeungnam University, Gyeongsan 38541, Republic of Korea; ashubahuguna@ynu.ac.kr (A.B.); vishalkumar@yu.ac.kr (V.K.); gabodkhe@yu.ac.kr (G.B.); sribt27@gmail.com (S.R.); thfvkalfpeh7@naver.com (S.L.); whdkfud12@naver.com (A.-r.J.); 2 Division of Food & Nutrition and Cook, Taegu Science University, Daegu 41453, Republic of Korea; jslee1213@ynu.ac.kr; 3 Department of Agrofood Resources, National Institute of Agricultural Sciences, Rural Development Administration, Wanju 55365, Republic of Korea; foodksy@korea.kr

**Keywords:** food safety, *Bacillus cereus*, biogenic amine, microbial analysis, fermentation, histamine, *doenjang*

## Abstract

The typical Korean diet contains a significant quantity of *doenjang* owing to its unique taste and health benefits. However, the presence of anti-nutritional and toxic substances, such as biogenic amines and microbial pathogens, in *doenjang* has resulted in a loss of revenue and poor consumer health. The present study focused on the identification and quantification of different biogenic amines, pathogenic *Bacillus cereus*, and yeast counts in 36 *doenjang* products (designated as De-1 to De-36, 500 g each) procured from the different cottage industries situated in different parts of the Republic of Korea. The results indicated, only three samples were contaminated with *B. cereus*, exceeding the recommended limit (4 log CFU/g) suggested by the national standards of Korea. A total of six distinct yeasts were identified in different *doenjang* samples, whose comprehensive enzymatic profiling suggested the absence of harmful enzymes such as N-acetyl-β-glucosaminidase, α-chymotrypsin, and β-glucuronidase. The biogenic amines were detected in the range of 67.68 mg/kg to 2556.68 mg/kg and classified into six major groups based on hierarchical cluster analysis. All *doenjang* samples contained tryptamine, putrescine, cadaverine, histamine, and tyramine, while 94.44% were positive for spermidine and spermine. The results documented the analysis of traditional cottage industry *doenjang* and suggest the need for constant monitoring to ensure the safety of food for the consumer.

## 1. Introduction

*Doenjang* is a famous traditional Korean fermented soybean paste that generally has a high salt content (18–20%) and is used in Korean cuisine as a source of nutrition and flavor [1,2]. *Doenjang* is gaining considerable attention worldwide because of its unique taste and functional activities, including anticancer, antioxidant, anti-obesity, anti-inflammatory, immunomodulatory, and fibrinolytic properties [3]. In a recent clinical study, the efficacy and safety of *doenjang* powder on gut microbiota and immunomodulation were documented (U.S. National Library of Medicine report). In 2020, the total gross national production of *doenjang* was 89,822 tons with a production value of KRW 114.4 billion [4].

*Doenjang* can be prepared in two different ways: traditionally at home or on a large industrial scale with strict quality control using a starter (*meju*) and brine solution (salt) as the main ingredients [5]. Household or cottage *doenjang* industries depend on traditional processes in which naturally occurring microorganisms participate and fermentation occurs in a natural environment. In contrast to this large-scale industry, *doenjang* is produced under strict fermentation conditions using pure starter cultures [5]. The *doenjang* produced by traditional and industrial methods differs greatly in terms of its nutritional value and organoleptic properties due to the involvement of varying native microflora, processing methods, and fermentation duration. Cottage-industry-prepared traditional *doenjang* is generally preferred by consumers over *doenjang* produced on a larger industrial scale owing to its unique flavor and taste [6]. Cottage industry and homemade *doenjang* are made from traditional *meju*. To prepare traditional *meju*, soybeans are boiled, crushed, and molded into a brick shape, which is then hung in the open for 1–2 months for natural fermentation. During this period, soybeans are fermented by natural microflora and are sometimes contaminated with pathogenic microorganisms [7]. Moreover, utensils and handling procedures used during *meju* preparation may also cause pathogenic contamination. Once the *meju* is formed, it is dipped in a high-salt solution for 2 months in closed earthen pots, and there is a low chance of microbial contamination. *Bacillus cereus*, owing to its halotolerant nature, is a matter of concern [8,9]. Despite the unique taste and numerous benefits of non-standardized cottage industry methods, they are often vulnerable to contamination by bacterial pathogens, mainly *B. cereus*, during natural fermentation. In addition to the presence of different anti-nutritional substances, toxicogenic biogenic amines also pose a threat to the consumption of cottage-industry-prepared *doenjang* [10]. Biogenic amines are decarboxylation products of amino acids and are listed as serious anti-nutritional and toxic agents at high concentrations [11]. Certain biogenic amines can react with nitrites to form the potent carcinogenic agent nitrosamine [12]. Indirectly, a high amount of biogenic amines correlates with microbial contamination and poor hygienic conditions in fermented foods. Biogenic amines, specifically histamines, are a major concern in food. Although there is no legal limit for histamines in food samples, a few reports have suggested that 100 mg/kg of histamine is toxic [13]. Similarly, in Korea and China, 250 mg/kg and 200–400 mg/kg of histamine, respectively, are the maximum allowable limits in fish [14,15,16]. Tyramine is another biogenic amine in food, and some reports have documented 100–800 mg/kg tyramine as the toxicity limit [13].

Therefore, monitoring biogenic amine levels in protein-rich fermented foods is essential for assessing food safety. To ensure the safety of *doenjang* for consumption and to control food pathogens and biogenic amine contamination, it is necessary to investigate these parameters in cottage industry *doenjang* products, which have not yet been investigated. Therefore, the present study is structured to evaluate the microbial count, precisely pathogenic *B. cereus* and yeast count, following enzymatic profiling, ethanol, and different biogenic amine quantifications in 36 *doenjang* products collected from cottage industries in the different provinces of South Korea. 

## 2. Materials and Methods

### 2.1. Chemicals

All chemicals used were of analytical grade. Standard biogenic amines [histamine (CAS No. 56-92-8), tyramine (CAS No. 60-19-5), putrescine (CAS No. 333-93-7), spermidine (CAS No. 334-50-9), tryptamine (CAS No. 343-94-2), spermine (CAS No. 306-67-2), 2-phenylethylamine (CAS No. 156-28-5), and cadaverine (CAS No. 1476-39-7)] were acquired from Sigma-Aldrich (St. Louis, MO, USA). The chemicals used for the extraction of biogenic amines, such as perchloric acid (CAS 7601-90-3), sodium hydroxide (CAS 1310-73-2), sodium hydrogen carbonate (CAS 144-55-8), dansyl chloride (CAS 605-65-2), ammonium acetate (CAS 631-61-8), acetonitrile (CAS 75-05-8), and ammonium hydroxide (CAS 1336-21-6), were analytical grade or HPLC (high performance liquid chromatography) grade and purchased from Duksan Pure Chemicals (Ansan-si, Republic of Korea).

### 2.2. Sample Collection

A total of 36 *doenjang* products (designated as De-1 to De-36, 500 g each) were procured from different cottage industries situated in different parts of South Korea, covering all provinces (Supplementary Table S1). The main ingredients of *doenjang* are steamed and milled soybean paste and sea salt. The samples were collected at the ambient temperature and stored at 4 °C for further analysis. The pH, salinity, color, and free amino nitrogen content of all the samples were examined (Supplementary Material M1–M3 and Supplementary Table S2).

### 2.3. Microbial Profile

#### 2.3.1. Isolation of Yeast, *B. cereus*, and Aerobic Bacteria

The standardized protocol established by the Association of Official Analytical Chemists in 1999 [17] was employed for the quantitative analysis of yeasts, *B. cereus*, and mesophilic/aerobic bacteria in the *doenjang* samples. Total yeast and mold, *B. cereus*, and aerobic bacteria were detected using 3 M Petrifilm (3 M; Health Care, St. Paul, MN, USA), mannitol egg yolk polymyxin agar medium (Oxoid, Hants, UK), and plate count agar medium (Becton Dickinson and Company, Franklin Lakes, NJ, USA), respectively. API 50CHB (bioMérieux, Marcy-l’Étoile, France) and API 20E kits (bioMérieux) were used to identify *B. cereus*. Potato dextrose agar (PDA) medium (Becton Dickinson and Company, NJ, USA) was used for yeast isolation.

#### 2.3.2. Molecular Identification of Yeast

Yeast isolated from *doenjang* was subjected to molecular characterization following the earlier described procedure with slight modification [18] using internal transcribed spacer ITS1 (5′-TCCGTAGGTGAACCTGCGG-3′) and ITS4 (5′-TCCTCCGCTTATTGATATGC-3′) primers (SolGent Co., Ltd., Daejeon, Republic of Korea) employing the following PCR conditions: 95 °C for 10 min (initial denaturation), 30 cycles at 95 °C for 1 min (denaturation), 55 °C for 2 min (annealing), 72 °C for 2 min (extension), and a final extension at 72 °C for 15 min [18]. The amplified fragments were sequenced using an ABI PRISM 3730XL DNA analyzer (Applied Biosystems, Foster City, CA, USA) by availing of a commercial facility of SolGent Co., Ltd. (Daejeon, Republic of Korea). The sequences of the microbes were aligned to the National Center for Biotechnology Information (NCBI) GenBank database using BLAST, and a phylogenetic tree was constructed using the neighbor-joining method with MEGA-6 software.

### 2.4. Enzyme Profiling of Isolated Yeasts

Enzyme profiling of the isolated yeasts was performed using an API ZYM kit (bioMérieux). Yeast cell suspensions (1 × 10^5^ cell/mL) were prepared in normal saline (0.85% NaCl). Sixty-five microliters of cell suspension were transferred to each well of the API ZYM strip and incubated at 37 °C for 4 h. After incubation, Zym A and Zym B were added, and enzyme activity was recorded based on color intensity on a scale of 0–5 and quantified as described by Kumar et al. [19]. A total of 19 enzymes were examined. A list of enzymes and their substrates is provided in Supplementary Table S3.

### 2.5. Analysis of Total Ethanol Content

The total ethanol content of *doenjang* was analyzed using a GC-MS QP2010 Ultra (Shimadzu, Kyoto, Japan) at the Core Research Support Center for Natural Products and Medical Materials at Yeungnam University, according to the protocol described by Gil et al. [20]. *Doenjang* (0.5 g) was extracted using dimethyl sulfoxide (9.5 mL) under constant stirring (100 rpm) at 40 °C for 1 h. The filtered supernatant of the *doenjang* extract was subjected to GC-MS. The operating GC-MS conditions were as follows: injector temperature, 160 °C; sample volume, 20 µL; split ratio, 40:1; oven temperature, initial 40 °C (5 min), increased to 240 °C by 10 °C/min, and isothermal 240 °C (9 min); ion-source, 70 eV, 200 °C; and scan intervals, 0.5 s. Ethanol (0.2%) acted as a reference to determine the ethanol quantity in the *doenjang* samples.

### 2.6. Estimation of Biogenic Amines

#### 2.6.1. Extraction of Biogenic Amines from *Doenjang*

Biogenic amines in *doenjang* were determined using the method described by Kumar et al. [21]. Twenty-five milliliters of perchloric acid (0.4 M) were used to extract around 5 g of *doenjang*, which was centrifuged at 4000× *g* for 10 min at 4 °C. The supernatant was collected and mixed with 200 µL of NaOH (2 M), 300 µL of saturated NaHCO_3_, and 2 mL of dansyl chloride (10 mg/mL), followed by a 45 min incubation at 40 °C [21]. Finally, 100 µL of NH_4_OH (25%) was added to the reaction mixture, and the volume was adjusted to 5 mL using acetonitrile, followed by centrifugation at 2500× *g* for 5 min. The supernatant was collected and passed through a 0.2 µm syringe filter (Sartorius AG, Goettingen, Germany).

#### 2.6.2. Quantification of the Biogenic Amines

An HPLC system (Thermo Fisher Scientific, Waltham, MA, USA) equipped with a C18 column (5 µm pore size, 0.46 × 25 cm, Waters Corporation, Milford, MA, USA) was used to quantify the biogenic amines. Twenty microliters of the sample were injected and eluted with 0.1 M ammonium acetate and acetonitrile in the mobile phase at a constant flow rate of 1 mL/min for 35 min at a constant column temperature of 40 °C [21]. The biogenic amines were detected at 254 nm. Standards of histamine, tyramine, putrescine, spermidine, tryptamine, spermine, 2-phenylethylamine, and cadaverine at 0–200 µg/mL concentrations were used to plot a standard curve that was used for the quantification of the respective biogenic amines in the sample.

### 2.7. Method Validation

The method validation involved generating a standard curve spanning various biogenic amine concentrations (Supplementary Figure S1) and the corresponding peak areas. The slope and the standard deviation intercept were calculated using the regression line to establish the limit of detection (LOD) and limit of quantification (LOQ). A known quantity (100 mg/kg) of individual biogenic amines was spiked in soybean paste and subsequently recovered following the method described in Section 2.6. The recovery percentage was calculated using the following formula: (concentration quantified in the sample using specified biogenic amines standard equation/original spiked concentration) × 100.

### 2.8. Statistical Evaluation

Triplicate experiments were performed, and the data are presented as the mean ± standard deviation (SD). Statistical differences between groups were determined by one-way analysis of variance (ANOVA) using Duncan’s multiple range test at *p* < 0.05 using the SPSS software (IBM, Chicago, IL, USA). A multivariate exploratory technique, principal component analysis (PCA), was performed to characterize and classify *doenjang* samples based on their biogenic amine levels using Minitab version 21.4 software.

## 3. Results and Discussion

### 3.1. Physicochemical Analysis 

The physiochemical assessment of all the *doenjang* samples was conducted, and the comprehensive findings are outlined in Supplementary Table S2. The highest pH was observed for De-34 (6.83 ± 0.02), whereas the least was observed for De-3 (4.65 ± 0.01) (Supplementary Table S2). The salinity of the tested *doenjang* samples was between 10.83% ± 0.00% and 16.79% ± 0.54% (Supplementary Table S2). The least salinity was observed in De-26 (10.83% ± 0.00%), whereas the highest was in De-35 (16.79% ± 0.54%). As an important physiochemical parameter, the color value of *doenjang* samples was assessed, and the observed lightness (L*) was in the range of 29.72 ± 0.15–46.32 ± 0.21; redness (a*), 3.63 ± 0.03–9.75 ± 0.02; and yellowness (b*), 4.12 ± 0.01–17.26 ± 0.08. Maximum and minimum values of L* were noticed for De-14 (46.32 ± 0.21) and De-2 (29.72 ± 0.15), respectively. The minimum a* (3.63 ± 0.03) and b* (4.12 ± 0.01) values were noticed for sample De-34, whereas the highest values were observed for De-30 (9.75 ± 0.02) and De-13 (17.26 ± 0.08), respectively (Supplementary Table S2). The amino nitrogen content in all the samples ranged from 210.42 ± 8.10 mg/100 g to 743.48 ± 21.43 mg/100 g. The maximum amino nitrogen content was quantified in De-18 (743.48 ± 21.43 mg/100 g), whereas the least was in De-3 (210.42 ± 8.10 mg/100 g) (Supplementary Table S2).

### 3.2. Microbial Profile Analysis

**Figure 1:**
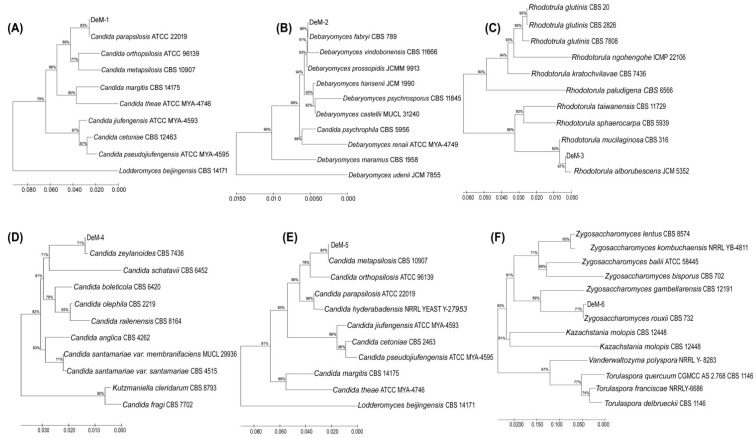
Comparative phylogenetic analysis of the yeast isolates from *doenjang* samples, (**A**) DeM-1, (**B**) DeM-2, (**C**) DeM-3, (**D**) DeM-4, (**E**) DeM-5, and (**F**) DeM-6. A phylogenetic tree was constructed using MEGA6.0 software by employing the neighbor-joining method.

Microbial analysis of fermented foods is highly recommended for examining product quality and safety parameters. Diverse microorganisms, either individually or in consortia, are used to prepare *doenjang* [5,22]. Given the key involvement of microorganisms in fermented food, we investigated their presence in *doenjang* samples. Initially, we examined the aerobic mesophilic bacteria and enumerated them at 8.92 ± 0.02 log CFU/g–2.54 ± 0.13 log CFU/g (Table 1). The aerobic bacteria count between the groups displayed a “df” value of 35 and a “F” value of 1542.6. The highest bacterial count was observed in De-21 (8.92 ± 0.02 log CFU/g), whereas the least was observed in De-10 (2.54 ± 0.13 log CFU/g). The majority of the samples (50.0%) had bacterial counts ranging from 5 log CFU/g to 8 log CFU/g. Only 5.56% and 44.44% of the samples had bacterial counts lower than 5 log CFU/g and higher than 8 log CFU/g, respectively. The aerobic bacterial count depends on the physicochemical environment of the *doenjang* and meju used as starter cultures. Furthermore, the bacterial population determines the final fate (aroma, color, and taste) of fermented foods, such as *doenjang*. In addition to bacteria, the active involvement of fungi and yeasts has been documented in *doenjang* [23]. All samples had yeast counts ranging from 2.18 ± 0.02 to 7.11 ± 0.04 log CFU/g (Table 1) with a “df” value of 35 and a “F” value of 841.7 between the groups. The highest yeast count was observed for De-26 (7.11 ± 0.04 log CFU/g), and the lowest for De-34 (2.18 ± 0.02 log CFU/g). Among the *doenjang* samples tested, 41.66% had a yeast count < 4 log CFU/g (Table 1). There are many reasons for the variation in yeast count among *doenjang* samples, but the choice of meju is by far the most significant. These findings support previous published reports that indicate the existence of various microorganisms in *doenjang* [13,14]. Furthermore, 43 yeast colonies were isolated from *doenjang* samples. Based on colony characteristics and microscopic examination, only six distinct yeast isolates (DeM-1, DeM-2, DeM-3, DeM-4, DeM-5, and DeM-6) were observed. The isolates were processed for molecular identification by ITS sequencing and comparative phylogenetic analysis, which revealed that DeM-1, DeM-2, DeM-3, DeM-4, DeM-5, and DeM-6 were the closest homologs of *Candida parapsilosis* ATCC 22019, *Debaryomyces fabryi* CBS 789, *Rhodotorula alborubescens* JCM 3552, *Candida zeylanoides* CBS 619, *Candida metapsilosis* CBS 10907, and *Zygosaccharomyces rouxii* CBS 732, respectively (Figure 1).

The sequences of the identified yeasts have been deposited in the NCBI GenBank with the allotted accession numbers DeM-1 (OQ729884), DeM-2 (OQ729885), DeM-3 (OQ729886), DeM-4 (OQ729887), DeM-5 (OQ729888), and DeM-6 (OQ729889). These findings offer a broad review of the bacterial counts and prevailing yeast strains in traditional *doenjang*, serving as a valuable reference for future research.

### 3.3. B. cereus Detection

From a food safety perspective, the presence of pathogenic bacteria is a serious concern. Every year, approximately 76 million ailments and numerous deaths occur worldwide due to food poisoning [24]. In general, the high salt concentration of food provides a barrier to most pathogenic microbes; however, several microbes, such as *B. cereus*, can grow efficiently in high-salt conditions [8,9] and are thus considered severe pathogens of fermented food products. In Europe, *B. cereus* is the third most common pathogen responsible for food poisoning after *Salmonella* and *Staphylococcus aureus* [25]. In the current study, 13.88% of the *doenjang* samples contained *B. cereus* (Table 1). The maximum number of *B. cereus* was observed in De-5 (6.15 log CFU/g), whereas the minimum number was observed in De-17 and De-29 (3.30 log CFU/g). Three samples (De-2, De-5, and De-19) were contaminated with *B. cereus* at levels above the recommended limit of 4 log CFU/g, as suggested by the national standard of Korea [26]. There is a high similarity between *B. cereus* and *B. thuringiensis*, which cannot be segregated by biochemical and molecular characterization based on 16S rRNA gene sequencing [27]. Therefore, to confirm that the isolates were *B. cereus* and not *B. thuringiensis*, crystal protein staining was performed as described by Bahuguna et al. [27] (Supplementary Material M4). None of the isolates displayed the formation of crystal proteins, a typical marker of *B. thuringiensis*, confirming that they were *B. cereus* and not *B. thuringiensis*.

*B. cereus* counts below the toxicity limit in most of the samples suggested that *doenjang* was hygienically prepared; however, a few samples raised concerns regarding *B. cereus* contamination. *B. cereus* contamination of *doenjang* cannot be attributed to a specific, discernible cause. However, the most likely reason for its presence is poor hygiene during preparation or the presence of *B. cereus* in the raw materials used for *doenjang* production. The present study’s outcomes were consistent with the findings of Park et al. [28], who tested the presence of *B. cereus* in 43 traditional *doenjang* samples and observed that 27.9% of the samples had *B. cereus* counts below 4 log CFU/g. In a similar study by Kim and Kim [29], *B. cereus* was detected in 18 homemade *doenjang* samples and revealed that 33.33% of the samples were positive for *B. cereus*, although only 22.22% of the samples had a *B. cereus* count higher than the suggested limit of 4 log CFU/g.

### 3.4. Enzymatic Profiling of Yeast Isolates

**Figure 2:**
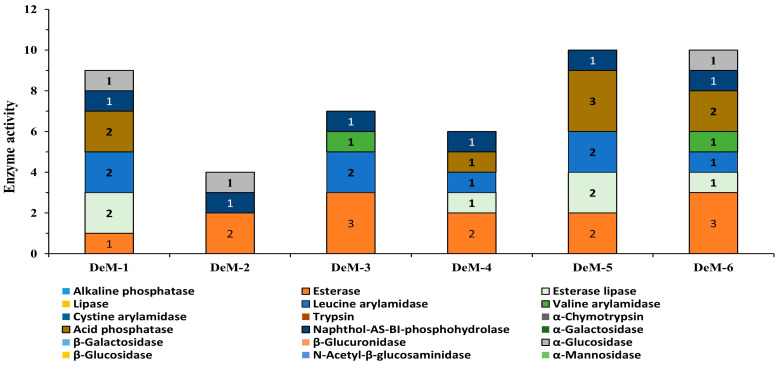
Enzymatic profiling of the yeasts isolated from *doenjang* samples. Enzyme activity 0 represents 0 nmol of product formed, while 1, 2, and 3 represent about 5 nmol, 10 nmol, and 20 nmol of product formed.

To ensure food safety, microorganisms used in the production of fermented foods must lack enzymes responsible for the generation of toxic metabolites [30]. In this study, yeasts isolated from different *doenjang* samples were comprehensively evaluated for enzyme activity using an API enzyme kit (Figure 2). Interestingly, all isolated strains produced naphthol-AS-BI-phosphohydrolase and esterase enzymes, which essentially contribute to the hydrolysis of aliphatic and aromatic esters into relevant acid forms and are commonly reported as flavor enhancers [31]. In addition, four isolates (DeM-1, DeM-4, DeM-5, and DeM-6) were positive for acid phosphatase, which causes digestion of phosphate from macromolecules, while three isolates (DeM-1, DeM-2, and DeM-6) were observed for the production of α-glucosidase, a key enzyme required to breakdown the oligo and disaccharides in intricate carbohydrates [32] (Figure 2). Most importantly, all isolates show negative activity for the enzymes linked with intestinal illnesses, such as β-glucuronidase, α-chymotrypsin, and N-acetyl-β-glucosaminidase [33]. Lipase, alkaline phosphatase, α-fucosidase, trypsin, cystine arylamidase, β-galactosidase, β-glucosidase, α-mannosidase, and α-galactosidase enzyme activity were negative in the isolates (Figure 2). The findings demonstrate the complete enzymatic profiling of the yeasts isolated from *doenjang* that do not possess any toxic enzymes for humans, thus verifying the nontoxic enzymatic nature of the isolates and advocating their application as food supplements.

### 3.5. Quantification of Ethanol

Ethanol is one of the chief volatile constituents of fermented foods and is often associated with their sensory properties [34]. However, high concentrations lead to detrimental sensory effects and discourage halal food guidelines [35]. Moreover, individuals deficient in acetaldehyde dehydrogenase accumulate large amounts of acetaldehyde that is metabolized from ethanol, which interferes with the DNA repair system [35]. Additionally, alcohol-food interactions are a serious concern, wherein high amounts of alcohol interact with food, alter its biological effects, and affect the absorption, storage, biotransformation, and excretion of vitamins B6, B12, and B9 [36]. The ethanol content of all *doenjang* samples ranged between 0% and 3.20% (Table 1). All *doenjang* samples, except De-14 (2.28%) and De-33 (3.20%), exhibited an alcohol content below 1%, which is the suggested limit for halal foods [35]. Ethanol quantification revealed a low amount of ethanol in *doenjang* samples; however, a few samples exhibited an abnormally high ethanol content. High salt concentrations may be a reason for the low ethanol content, as high salt concentrations represent a preventive barrier for many alcohol-producing yeasts such as *Saccharomyces cerevisiae*. Moreover, the variation in ethanol content in *doenjang* samples is due to the diversification of microorganisms, particularly yeasts, which are the major producers of alcohol in fermented foods [37]. The results of this study are in agreement with those of many previously published studies reporting similar ethanol profiles in *doenjang* samples [38,39]. Low-to-moderate ethanol content has an impact on the unique flavor of *doenjang* and favors its trade in Muslim countries where halal certification is required.

### 3.6. Biogenic Amines

**Figure 3:**
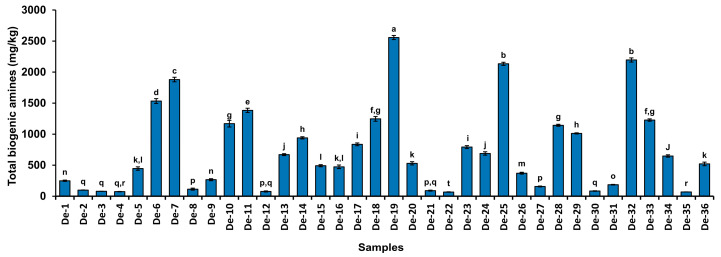
Total biogenic amines in 36 cottage industry *doenjang*. The bar diagram values represent the mean value of the two independent experiments, and the different letters above the bar diagram represent a significant (*p* < 0.05) difference between the groups. The “df” between the groups is 35, and the “F” value is 1671.8.

Biogenic amines are regarded as toxicants and anti-nutritional components of food that arise from amino acids through decarboxylation reactions catalyzed by microorganisms [11]. Excessive amounts of biogenic amines have several detrimental effects, including dizziness, headaches, cardiac palpitations, respiratory illnesses, hypertension, and hypotension [40]. Furthermore, various biogenic amines react with nitrite to form nitrosamine, which is a potent carcinogenic agent [12]. Numerous factors, including microbes and food composition, are responsible for the production of biogenic amines in food. *Doenjang* is a protein-rich food that contains a variety of free amino acids [41]. Free amino acids are precursors for the synthesis of biogenic amines through the action of microbial decarboxylases [11]. Hence, the evaluation of biogenic amines is crucial for food safety, particularly in protein-rich foods. Total biogenic amines in all *doenjang* samples were in the range of 67.68 ± 3.06 mg/kg to 2556.68 ± 33.0 mg/kg (Figure 3, Table 2), with a ‘df’ value of 35 and a “F” value of 1671.8 between groups. Sample De-19 was detected with the highest concentration of total biogenic amines (2556.68 ± 33.0 mg/kg), whereas De-22 had the lowest concentration (67.68 ± 3.06 mg/kg). (Figure 3, Table 2). Although no rigorous recommendations exist for total biogenic amines in fermented foods, a few reports have suggested that total biogenic amines up to 1000 mg/kg are permissible [42]. Biogenic amines were within the limit in the majority of tested samples (69.44%), which agrees with published reports showing similar profiles of biogenic amines in different *doenjang* [43,44,45].

**Figure 4:**
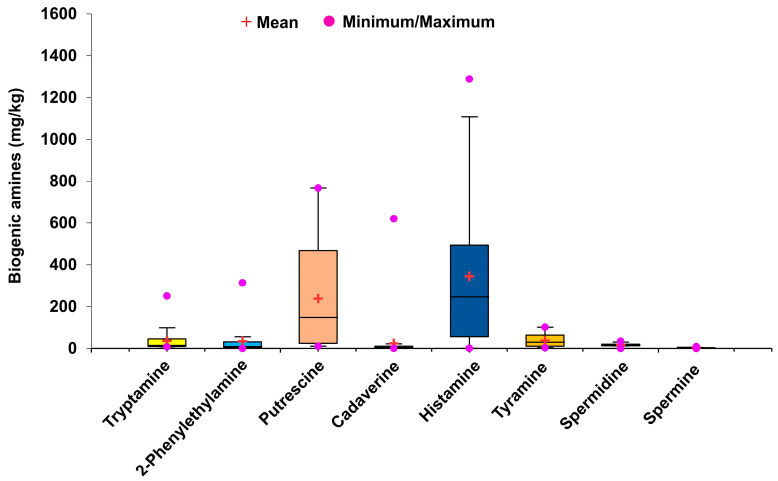
Mean values and minimum and maximum amounts of different biogenic amines in 36 cottage industry *doenjang* samples.

Among the various types of biogenic amines, histamine is considered to be the most severe toxicant responsible for poisoning (scombroid poisoning). Several studies have suggested toxic effects of histamine at concentrations higher than 500 ppm [11]. Although no strict guidelines exist for the histamine level in fermented food samples, a few reports suggest 100 mg/kg histamine as the toxic limit [13]; however, the World Health Organization suggests a maximum permissible limit of 200 mg/kg histamine for the consumption of fish and fish products [46]. Similarly, the maximum allowable limits for histamine in fish set by Korea and China are 250 mg/kg and 200–400 mg/kg, respectively [14,15,16]. Histamine was present in all *doenjang* samples in the range 0.97 ± 0.04–1289.02 ± 11.23 mg/kg (Figure 4 and Table 2). Nine *doenjang* samples (De-6, De-7, De-10, De-14, De-18, De-19, De-25, De-29, and De-32) had abnormally high histamine levels, higher than the suggested toxic limit of 500 ppm [11]. The results are in accordance with earlier reports suggesting a variable histamine level in different *doenjang* samples [43,44,47]. Similar to this, Bahuguna et al. [41] demonstrated the presence of varying amounts of histamine in different *doenjang* samples.

Tyramine is another significant biogenic amine, whose excess leads to symptoms similar to those of histamine poisoning [11]. The tyramine level in the *doenjang* samples was between 3.23 ± 0.09 mg/kg and 101.23 ± 2.53 mg/kg (Figure 4 and Table 2). The minimum and maximum tyramine levels were detected in De-3 (3.23 ± 0.09 mg/kg) and De-11 (101.23 ± 2.53 mg/kg) samples, respectively. The tyramine levels in 97.22% of *doenjang* samples were below the suggested tyramine toxicity level of 100–800 mg/kg in food [13]. These results are in accordance with the findings of Bahuguna et al. [41], who reported similar amounts of tyramine in 10 garlic-supplemented *doenjang* samples. Putrescine, spermidine, and spermine are other biogenic amines that frequently exist in foods and have 2000 mg/kg, 600 mg/kg, and 600 mg/kg limits of oral toxicity, respectively [11]. All tested *doenjang* samples show significantly varying amounts of putrescine (10.79 ± 0.10 to 767.03 ± 13.09 mg/kg), spermidine (0 to 34.29 ± 1.62 mg/kg), and spermine (0 to 8.49 ± 0.11 mg/kg) (Figure 4 and Table 2). All samples contained putrescine, spermidine, and spermine levels below their oral toxicity limits [11]. These findings were corroborated by published reports demonstrating variations in these biogenic amines in different *doenjang* samples [43]. Similarly, Kim et al. [44] detected putrescine and spermidine in seven *doenjang* samples; however, spermine was not detected in any sample.

The significant contribution of 2-phenylethylamine to food-induced migraines and hypertension has been cited in the literature [11]. Some studies have reported that 30 mg/kg of 2-phenylethylamine is an acceptable limit for common foods [40]. In the tested *doenjang* samples, 2-phenylethylamine levels ranged from 0 mg/kg to 313.56 ± 4.41 mg/kg (Figure 4 and Table 2). Overall, 72.2% of the samples had 2-phenylethylamine levels lower than the suggested 30 mg/kg [40], which is consistent with reports revealing a similar range in different *doenjang* samples [43,44,47].

The toxigenic role of tryptamine has been previously described by the European Food Safety Authority. A high concentration of tryptamine may cause vasocontraction, leading to high blood pressure, vomiting, swelling, and headaches [48]. Moreover, tryptamine can enhance histamine toxicity by inhibiting the histamine-destroying enzyme, diamine oxidase. Currently, no legal limits have been established for tryptamine. However, some studies have suggested that consumption of tyramine at 480 mg/day has no adverse health effect [48]. The present study revealed the tryptamine value in the range of 8.03 ± 0.03 mg/kg to 251.07 ± 3.02 mg/kg (Figure 4 and Table 2). Only four samples, De-6 (111.86 ± 4.61 mg/kg), De-18 (251.07 ± 3.02 mg/kg), De-25 (123.04 ± 1.90 mg/kg), and De-33 (101.41 ± 3.88 mg/kg), had tryptamine levels higher than 100 mg/kg.

Several factors, such as the types of raw materials, environmental conditions, fermentation time, and manufacturing practices, may be responsible for the differences in biogenic amine levels in fermented foods [49]. However, the microbial population and physiochemical conditions are the most influential factors responsible for biogenic amine production in different samples [49]. Different strains of *Bacillus* sp. are the dominant bacterial species during *doenjang* fermentation [10,50]. Several reports have suggested the role of *B. subtilis*, *B. licheniformis*, and *B. amyloliqueformis* as producers of biogenic amines, owing to their decarboxylase activity [49]. The presence of these strains in *doenjang* is a major cause of biogenic amine production. The activity between the strains may vary, which is one of the principal reasons for the differences in biogenic amine levels among the samples. Furthermore, a few strains of *Bacillus* spp. have been found to be effective at degrading biogenic amines [40,51]. *Bacillus* sp. is the predominant bacterial species in *doenjang* and thus may be responsible for the variation in biogenic amine levels in the tested *doenjang* samples. Due to the above-mentioned reason, a wide range of tryptamine (8.31–251.07 mg/kg), 2-phenylethylamine (0–313.56 mg/kg), putrescine (10.79–767.03 mg/kg), histamine (0.97–1289.02 mg/kg), tyramine (3.23–101.23 mg/kg), spermidine (0–34.29 mg/kg), and spermine (0–8.49 mg/kg) were detected in the tested 36 cottage industry *doenjang* (Figure 4).

Collectively, these results indicate that most of the tested *doenjang* samples possessed biogenic amine contents below the toxicity threshold suggested by numerous regulatory bodies. However, a few samples had a biogenic amine content higher than the suggested value. This study offers a wide spectrum of biogenic amines commonly found in cottage industry *doenjang* and serves as a valuable reference for assessing the biogenic profile of *doenjang*.

### 3.7. Multivariate Investigation

**Figure 5:**
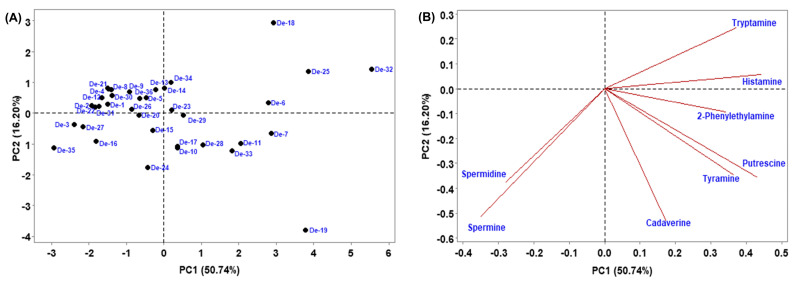
Principal component (PC) analysis of biogenic amines in *doenjang* samples. (**A**) PC1 and PC2 score plots, and (**B**) PC1 and PC2 loading.

**Figure 6:**
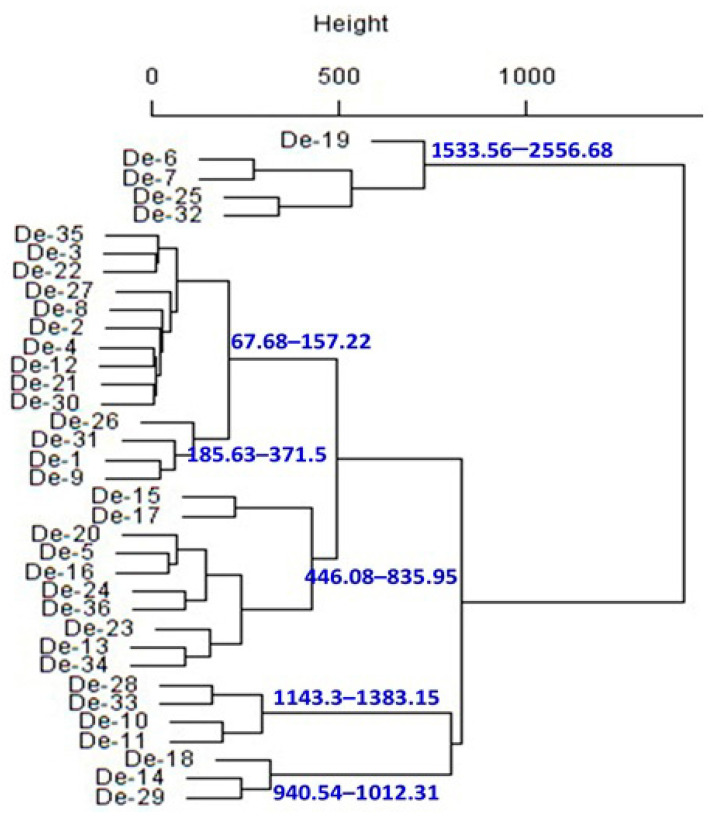
Dendrogram obtained using classical cluster analysis for the mean of biogenic amines for each *doenjang* sample.

To make the investigation easier, principal component analysis (PCA) was used to determine how multivariate data related to smaller dimensions. Multivariate analysis of biogenic amines indicates that the 2-principal component (PC) model explains 66.94% of the variance (Figure 5). PC1 explained 50.74% of the variance and separated De-6, De-7, De-10, De-11, De-13, De-14, De-18, De-19, De-23, De-25, De-28, De-29, De-32, De-33, and De-34 (PC1 values positive) from the rest of the samples (Figure 5A). PC2 shows 16.20% of variance and segregated De-4, De-5, De-6, De-8, De-9, De-12, De-13, De-15, De-21, De-22, De-23, De-25, De-26, De-30, De-31, De-32, De-34, and De-36 (positive PC2 values) from the other samples (Figure 5A), principally due to tryptamine and histamine (positive values) (Figure 5B). Furthermore, hierarchical cluster analysis (HCA), which is regarded as a powerful technique to build a tree based on the similarity between a group of large datasets, was performed. The HCA dendrogram separated the 36 *doenjang* samples into different clusters of the biogenic amine (Figure 6). *Doenjang* samples De-6, De-7, De-25, and De-32 show similar distances from their cluster centers, indicating similar profiles of biogenic amines. Similarly, *doenjang* samples (De-2, De-4), (De-3, De-22), (De-21, De-30), (De-1, De-4), (De-5, De-16), (De-24, De-36), (De-13, De-34), (De-28, De-33), (De-10, De-11), and (De-14, De-19) show similar distances from their cluster centers, indicating similar biogenic amine profiles. Based on the classical cluster analysis findings, we broadly classified the 36 *doenjang* samples into six major categories (Figure 6).

### 3.8. Method Validation

The linearity (*R*^2^ = 0.9891–0.9999) of the calibration curves plotted between the concentration of biogenic amines vs. the peak area suggested excellent linearity (Supplementary Figure S1 and Table S4). The LOD and LOQ for tryptamine, 2-phenylethylamine, putrescine, cadaverine, histamine, tyramine, spermidine, and spermine fell within the ranges of 0.10–0.30, 0.09–0.28, 0.34–1.03, 0.20–0.62, 0.72–2.18, 0.30–0.91, 0.44–1.34, and 0.99–3.02, respectively (Table S4). Furthermore, the recovery of biogenic amines in *doenjang* spanned from 75% to 82%.

## 4. Conclusions

In conclusion, the 36 *doenjang* samples showed diverse microbiological, alcohol, and biogenic amine profiles. Six distinct yeast isolates were identified in different *doenjang* samples that did not produce any harmful enzymes, indicating their safety for consumers. Five *doenjang* samples were contaminated with *B. cereus*; however, only three had *B. cereus* counts higher than the recommended limit of 4 log CFU/g. The ethanol contents of two *doenjang* samples were higher than the recommended limit of halal food guidelines (<1%). The biogenic amine levels in most *doenjang* samples were below the suggested limit. However, high amounts of biogenic amines were detected in only a few samples. These results collectively suggest that most *doenjang* samples are safe and consumable, except for a few samples containing *B. cereus* and biogenic amines. This study recommends conducting regular assessments of cottage industry products, especially in cases where quality control standards are less stringent than those in large-scale *doenjang* industries, to ensure product safety. Also, it is advisable to carry out these analyses in the very large sample size to establish a strong concluding remark.

## Figures and Tables

**Table 1:** Total aerobic bacteria, yeast count, and ethanol content of *doenjang* samples.

Sample Code	Aerobic Bacteria	Yeast	*Bacillus cereus*	Ethanol (%)
	Log Colony Forming Unit/g (Log CFU/g)			
De-1	8.52 ± 0.10 ^b^	4.42 ± 0.071 ^j^	ND ^(^*^)^	0.01
De-2	8.48 ± 0.04 ^b^	4.01 ± 0.08 ^l^	5.89 ± 0.0	0.02
De-3	6.06 ± 0.24 ^s^	2.73 ± 0.04 ^r^	ND	0.09
De-4	7.77 ± 0.26 ^lm^	5.83 ± 0.03 ^d^	ND	0.02
De-5	8.14 ± 0.09 ^ef^	4.68 ± 0.02 ^h^	6.15 ± 0.0	0.11
De-6	7.74 ± 0.22 ^l^	4.83 ± 0.01 ^g^	ND	0.02
De-7	7.82 ± 0.15 ^i^	2.90 ± 0.04 ^q^	ND	0.09
De-8	7.15 ± 0.11 ^p^	3.00 ± 0.04 ^pq^	ND	0.02
De-9	8.19 ± 0.09 ^de^	4.84 ± 0.03 ^g^	ND	0.08
De-10	2.54 ± 0.13 ^v^	3.78 ± 0.09 ^m^	ND	0.04
De-11	8.09 ± 0.11 ^h^	4.51 ± 0.03 ^ij^	ND	0.03
De-12	7.94 ± 0.10 ^j^	3.24 ± 0.05 ^o^	ND	0.01
De-13	7.43 ± 0.14 ^o^	6.65 ± 0.02 ^b^	ND	0.07
De-14	7.14 ± 0.14 ^r^	4.52 ± 0.02 ^ij^	ND	2.28
De-15	8.06 ± 0.06 ^g^	3.20 ± 0.14 ^o^	ND	0.03
De-16	8.48 ± 0.15 ^c^	3.77 ± 0.01 ^m^	ND	0.03
De-17	8.34 ± 0.07 ^cd^	4.61 ± 0.07 ^hi^	3.30 ± 0.0	0.03
De-18	6.64 ± 0.32 ^t^	3.05 ± 0.07 ^p^	ND	0.05
De-19	7.69 ± 0.19 ^lm^	3.70 ± 0.01 ^m^	4.15 ± 0.0	0.03
De-20	8.58 ± 0.15 ^b^	4.84 ± 0.05 ^g^	ND	0.04
De-21	8.92 ± 0.02 ^a^	5.04 ± 0.01 ^e^	ND	0.03
De-22	8.27 ± 0.06 ^ef^	4.96 ± 0.02 ^fg^	ND	0.08
De-23	7.64 ± 0.20 ^mn^	3.19 ± 0.05 ^o^	ND	0.06
De-24	8.40 ± 0.07 ^c^	6.08 ± 0.01 ^c^	ND	0.12
De-25	7.66 ± 0.16 ^l^	3.92 ± 0.02 ^l^	ND	0.17
De-26	7.69 ± 0.20 ^l^	7.11 ± 0.04 ^a^	ND	0.04
De-27	5.46 ± 0.04 ^q^	6.75 ± 0.07 ^b^	ND	0.19
De-28	7.94 ± 0.09 ^i^	5.03 ± 0.01 ^f^	ND	0.02
De-29	7.86 ± 0.17 ^k^	3.45 ± 0.04 ^n^	3.30 ± 0.0	0.00
De-30	8.23 ± 0.06 ^ef^	2.91 ± 0.17 ^q^	ND	0.07
De-31	8.94 ± 0.02 ^a^	6.77 ± 0.03 ^b^	ND	0.00
De-32	7.22 ± 0.16 ^q^	4.04 ± 0.10 ^l^	ND	0.12
De-33	8.34 ± 0.15 ^f^	2.88 ± 0.14 ^q^	ND	3.20
De-34	4.84 ± 0.53 ^u^	2.18 ± 0.02 ^s^	ND	0.24
De-35	8.13 ± 0.08 ^g^	4.95 ± 0.02 ^fg^	ND	0.01
De-36	7.36 ± 0.16 ^no^	4.28 ± 0.04 ^k^	ND	0.01

Values representing aerobic bacteria, yeast, and *Bacillus cereus* counts are based on the mean values of three independent experiments. Different letters represent the statistical difference between the samples (in columns) at *p* < 0.05. For aerobic bacteria and yeast count, the “df” between the groups is 35 and the “F” value is 1542.6 and 841.7, respectively. * ND: not detected.

**Table 2:** Quantification of biogenic amines (mg/kg) in 36 cottage industry *doenjang* samples.

Sample Code	Tryptamine	2-Phenylethylamine	Putrescine	Cadaverine	Histamine	Tyramine	Spermidine	Spermine	Total Biogenic Amine
**De-1**	14.61 ± 0.88	3.38 ± 0.4	29.13 ± 0.66	1.33 ± 0.08	165.14 ± 8.77	10.69 ± 0.17	19.23 ± 0.23	5.52 ± 0.16	249.04 ± 10.98 ^n^
**De-2**	9.61 ± 0.3	3.07 ± 0.02	23.22 ± 0.44	4.76 ± 0.12	17.31 ± 0.66	11.82 ± 0.33	23.82 ± 0.38	4.81 ± 0.04	98.41 ± 2.28 ^q^
**De-3**	8.03 ± 0.05	3.21 ± 0.04	10.79 ± 0.10	1.71 ± 0.21	14.28 ± 0.21	3.23 ± 0.09	32.02 ± 0.42	5.94 ± 0.19	79.22 ± 1.32 ^q^
**De-4**	8.73 ± 0.03	3.75 ± 0.05	13.79 ± 0.38	1.98 ± 0.09	23.61 ± 2.06	3.81 ± 0.02	13.12 ± 0.05	4.92 ± 0.01	73.70 ± 2.68 ^q,r^
**De-5**	21.46 ± 0.95	16.96 ± 0.77	114.10 ± 7.95	3.91 ± 0.41	240.48 ± 14.03	32.36 ± 2.97	12.07 ± 0.04	4.75 ± 0.05	446.08 ± 27.16 ^k,l^
**De-6**	111.86 ± 4.61	14.20 ± 3.72	540.96 ± 25.22	6.95 ± 0.11	763.58 ± 1.49	91.41 ± 3.34	0.00 ± 0.00	4.60 ± 0.03	1533.56 ± 38.53 ^d^
**De-7**	74.68 ± 4.96	232.53 ± 3.88	681.32 ± 5.58	22.10 ± 3.70	805.09 ± 16.82	44.04 ± 1.81	15.87 ± 0.28	4.66 ± 0.00	1880.29 ± 37.03 ^c^
**De-8**	8.99 ± 0.02	5.49 ± 0.12	19.32 ± 1.40	4.60 ± 5.14	42.23 ± 5.32	14.66 ± 0.35	13.82 ± 3.18	5.08 ± 0.68	114.19 ± 16.22 ^p^
**De-9**	11.08 ± 3.69	10.08 ± 0.45	38.20 ± 1.45	1.99 ± 0.35	165.23 ± 8.90	22.66 ± 1.64	12.23 ± 0.05	4.70 ± 0.02	266.19 ± 16.55 ^n^
**De-10**	18.34 ± 0.22	55.26 ± 4.76	485.64 ± 3.94	14.52 ± 3.73	520.83 ± 7.14	48.46 ± 3.83	20.21 ± 1.15	6.25 ± 0.35	1169.53 ± 53.27 ^g^
**De-11**	62.13 ± 1.85	35.01 ± 2.10	657.42 ± 11.84	24.42 ± 2.95	485.48 ± 12.72	101.23 ± 2.53	12.73 ± 0.07	4.73 ± 0.02	1383.15 ± 34.09 ^e^
**De-12**	8.12 ± 0.03	3.92 ± 0.13	13.44 ± 0.23	1.47 ± 0.13	24.28 ± 7.58	6.99 ± 0.23	13.37 ± 0.07	5.74 ± 0.18	77.34 ± 8.58 ^p,q^
**De-13**	42.30 ± 4.22	5.30 ± 1.64	135.00 ± 3.70	1.77 ± 0.52	448.11 ± 4.93	20.48 ± 0.10	12.19 ± 0.10	4.74 ± 0.05	669.89 ± 15.27 ^j^
**De-14**	16.40 ± 0.43	3.76 ± 0.30	49.18 ± 2.96	0.69 ± 0.04	827.81 ± 13.38	26.04 ± 0.10	12.01 ± 0.09	4.65 ± 0.02	940.54 ± 17.31 ^h^
**De-15**	12.49 ± 0.22	28.99 ± 3.66	264.63 ± 6.68	5.02 ± 0.26	93.29 ± 1.69	63.90 ± 4.10	17.84 ± 0.41	5.18 ± 0.02	491.33 ± 17.04 ^l^
**De-16**	13.84 ± 0.06	5.30 ± 0.05	143.76 ± 8.21	4.11 ± 0.16	252.97 ± 16.36	16.10 ± 0.83	30.71 ± 0.18	7.00 ± 2.80	473.80 ± 28.66 ^k,l^
**De-17**	54.54 ± 4.22	31.70 ± 3.46	462.37 ± 7.47	12.20 ± 1.12	182.45 ± 3.25	71.19 ± 3.12	14.69 ± 0.24	6.79 ± 0.17	835.95 ± 23.04 ^i^
**De-18**	251.07 ± 3.02	15.44 ± 1.41	223.33 ± 1.86	3.24 ± 0.13	710.87 ± 29.03	28.74 ± 2.00	13.65 ± 0.02	0.00 ± 0.00	1246.34 ± 37.47 ^f,g^
**De-19**	74.14 ± 2.89	79.36 ± 3.04	767.03 ± 13.09	620.33 ± 8.96	927.54 ± 3.64	70.41 ± 1.32	13.20 ± 0.06	4.67 ± 0.01	2556.68 ± 33.0 ^a^
**De-20**	13.95 ± 0.10	8.17 ± 0.08	153.39 ± 1.77	2.11 ± 0.04	288.14 ± 23.38	39.16 ± 0.57	20.32 ± 0.22	4.88 ± 0.06	530.12 ± 26.21 ^k^
**De-21**	11.28 ± 0.04	4.90 ± 0.39	19.49 ± 0.37	1.86 ± 0.10	24.69 ± 6.37	9.04 ± 0.17	13.97 ± 0.11	4.74 ± 0.02	89.95 ± 7.56 ^p,q^
**De-22**	9.15 ± 0.43	2.99 ± 0.03	11.35 ± 0.41	1.08 ± 0.04	10.92 ± 0.13	5.13 ± 1.69	21.58 ± 0.24	5.48 ± 0.08	67.68 ± 3.06 ^t^
**De-23**	14.60 ± 0.13	40.90 ± 2.97	270.16 ± 1.84	6.60 ± 0.15	400.82 ± 16.49	41.85 ± 0.53	12.85 ± 0.04	4.65 ± 0.01	792.42 ± 22.15 ^i^
**De-24**	13.42 ± 0.21	23.50 ± 1.66	238.86 ± 5.12	26.93 ± 1.05	254.81 ± 14.93	91.37 ± 2.76	34.29 ± 1.62	5.51 ± 0.07	688.68 ± 27.42 ^j^
**De-25**	123.04 ± 1.90	31.62 ± 0.94	602.36 ± 8.76	10.30 ± 0.22	1289.02 ± 11.23	62.54 ± 1.83	14.93 ± 0.43	0.00 ± 0.00	2133.80 ± 25.31 ^b^
**De-26**	10.22 ± 0.25	19.91 ± 0.47	104.63 ± 5.41	2.82 ± 0.04	180.43 ± 6.88	33.16 ± 1.42	14.93 ± 0.61	5.40 ± 0.07	371.50 ± 15.15 ^m^
**De-27**	10.28 ± 0.04	3.88 ± 0.04	38.13 ± 1.07	2.77 ± 0.08	60.14 ± 4.93	9.76 ± 0.17	25.39 ± 0.67	6.87 ± 0.32	157.22 ± 7.32 ^p^
**De-28**	14.95 ± 0.41	109.11 ± 5.10	534.80 ± 3.94	21.07 ± 0.45	376.07 ± 4.35	63.30 ± 2.00	19.19 ± 0.27	4.81 ± 0.01	1143.30 ± 16.52 ^g^
**De-29**	19.75 ± 0.52	10.57 ± 0.59	222.43 ± 1.25	4.58 ± 0.36	672.17 ± 3.51	63.22 ± 2.91	14.85 ± 0.22	4.74 ± 0.02	1012.31 ± 9.38 ^h^
**De-30**	9.01 ± 0.06	2.83 ± 0.01	22.80 ± 0.77	1.22 ± 0.04	24.92 ± 2.66	4.75 ± 0.11	13.26 ± 0.23	4.93 ± 0.04	83.72 ± 3.91 ^q^
**De-31**	9.14 ± 0.01	0.00 ± 0.00	25.20 ± 0.88	5.41 ± 0.28	112.29 ± 2.86	3.55 ± 0.05	24.96 ± 0.92	5.08 ± 0.06	185.63 ± 5.10 ^o^
**De-32**	98.36 ± 2.67	313.56 ± 4.41	599.66 ± 4.60	3.54 ± 0.15	1107.47 ± 17.47	73.02 ± 4.65	0.00 ± 0.00	0.00 ± 0.00	2195.62 ± 33.94 ^b^
**De-33**	101.41 ± 3.88	83.88 ± 1.60	626.29 ± 5.47	12.94 ± 2.02	285.30 ± 2.47	94.26 ± 1.89	17.22 ± 0.12	5.77 ± 1.47	1227.05 ± 18.91 ^f,g^
**De-34**	17.95 ± 1.77	3.97 ± 0.30	193.90 ± 4.29	4.99 ± 0.49	391.59 ± 8.90	31.02 ± 2.74	0.00 ± 0.00	4.68 ± 0.01	648.11 ± 18.49 ^j^
**De-35**	8.04 ± 0.03	2.70 ± 0.02	12.43 ± 0.28	0.85 ± 0.03	0.97 ± 0.04	3.45 ± 0.03	32.11 ± 0.19	8.49 ± 0.11	69.03 ± 0.73 ^r^
**De-36**	8.17 ± 0.01	3.44 ± 0.09	229.48 ± 6.70	14.73 ± 2.76	235.16 ± 18.57	12.71 ± 0.61	12.36 ± 0.13	4.65 ± 0.01	520.71 ± 28.88 ^k^

Values represent the mean value of the two independent experiments. Different letters represent the statistical difference between the total biogenic amines among the samples (in column) at *p* < 0.05. The “df” between the groups is 35, and the “F” value is 1671.8.

## Data Availability

Data is contained within the article.

## Supplementary Materials

The following supporting information can be downloaded at https://www.mdpi.com/article/10.3390/foods12224084/s1, Table S1: Details of the selected *doenjang* samples; Table S2: pH, salinity, color values, and amino nitrogen content of *doenjang* samples; Table S3: Enzymes and their substrates; Table S4: Limit of detection (LOD) and limit of quantification (LOQ) for different biogenic amines; Figure S1: Standard curves of different biogenic amines; Method M1: Determination of pH and salinity; Method M2: Determination of pH and salinity; Method M3: Determination of free amino nitrogen; Method M4: Crystalline protein staining.

## References

[1] Jeon H.H., Jung J.Y., Chun B.H., Kim M.-D., Baek S.Y., Moon J.Y., Yeo S.-H., Jeon C.O. (2016). Screening and characterization of potential *Bacillus* starter cultures for fermenting low-salt soybean paste (*Doenjang*). J. Microbiol. Biotechnol..

[2] Shin D., Jeong D. (2015). Korean traditional fermented soybean products: *Jang*. J. Ethn. Foods..

[3] Chun B.H., Kim K.H., Jeong S.E., Jeon C.O. (2020). The effect of salt concentrations on the fermentation of *doenjang*, a traditional Korean fermented soybean paste. Food Microbiol..

[4] Food Information Statistics System Processed Food Market Status (*Doenjang*) 2021. https://www.atfis.or.kr/home/board/FB0027.do?act=read&subSkinYn=N&bpoId=4135&bcaId=0&pageIndex=2.

[5] Shukla S., Lee J.S., Park H.-K., Yoo J.-A., Hong S.-Y., Kim J.-K., Kim M. (2015). Effect of novel starter culture on reduction of biogenic amines, quality improvement, and sensory properties of *Doenjang*, a traditional Korean soybean fermented sauce variety. J. Food Sci..

[6] Kim K.M., Lee K.-G. (2018). Defining gu-soo perception in *doenjang* (fermented soybean paste) using consumer tests with limited sensory modality and instrumental analysis. Food Chem..

[7] Lee E.J., Hyun J., Choi Y.H., Hurh B.S., Choi S.H., Lee I. (2018). Development of safe and flavor-rich *Doenjang* (Korean Fermented Soybean Paste) using autochthonous mixed starters at the pilot plant scale. J. Food Sci..

[8] Singh S.K., Tripathi V.R., Jain R.K., Vikram S., Garg S.K. (2010). An antibiotic, heavy metal resistant and halotolerant *Bacillus cereus* SIU1 and its thermoalkaline protease. Microb. Cell Factories.

[9] Wang P., Chen X.T., Qiu Y.Q., Liang X.F., Cheng M.M., Wang Y.J., Ren L.H. (2020). Production of polyhydroxyalkanoates by halotolerant bacteria with volatile fatty acids from food waste as carbon source. Appl. Biochem. Biotechnol..

[10] Jeong S.J., Ryu M.S., Yang H.J., Wu X.H., Jeong D.Y., Park S.M. (2021). Bacterial distribution, biogenic amine contents, and functionalities of traditionally made *doenjang*, a long-term fermented soybean food, from different areas of Korea. Microorganisms.

[11] Naila A., Finlt S., Fletcher G., Bremer P., Meerdink G. (2010). Control of biogenic amines in food-existing and emerging approaches. J. Food Sci..

[12] Kim M.K., Mah J.H., Hwang H.J. (2009). Biogenic amine formation and bacterial contribution in fish, squid and shellfish. Food Chem..

[13] Ten Brink B., Damink C., Joosten H.M.L.J., In’t Veld J.H. (1990). Occurrence and formation of biologically active amines in foods. Int. J. Food Microbiol..

[14] The Ministry of Food and Drug Safety (MFDS) (2017). Food Code.

[15] (2016). National Food Safety Standards for Fresh and Frozen Animal Aquatic Products.

[16] Park Y.K., Lee J.H., Mah J.-H. (2019). Occurrence and reduction of biogenic amines in traditional Asian fermented soybean foods: A review. Food Chem..

[17] Association of Official Analytical Chemists (AOAC) (1999). Official Methods of Analysis. Official Methods of Analysis.

[18] Li S.S., Cheng C., Li Z., Chen J.Y., Yan B., Han B.Z., Reeves M. (2010). Yeast species associated with wine grapes in China. Int. J. Food Microbiol..

[19] Kumar V., Bahuguna A., Lee J.S., Sood A., Han S.S., Chun H.S., Kim M. (2023). Degradation mechanism of aflatoxin B1 and aflatoxin G1 by salt tolerant *Bacillus albus* YUN5 isolated from ‘*doenjang*’, a traditional Korean food. Food Res. Int..

[20] Gil N.Y., Kim S.Y., Choi H.S., Park S.Y., Kim J.H. (2016). Investigation of quality characteristics and alcohol content in commercial Korean fermented sources. Korean J. Food Preserv..

[21] Kumar V., Bahuguna A., Kim M. (2022). Simultaneous detection and quantification of different biogenic amines. Bangladesh J. Pharmacol..

[22] Jeong D.-W., Jeong K., Lee H., Kim C.-T., Heo S., Oh Y., Heo G., Lee J.-H. (2020). Effects of *Enterococcus faecium* and *Staphylococcus succinus* starters on the production of volatile compounds during *doenjang* fermentation. LWT-Food Sci. Technol..

[23] Lee S., Lee S., Singh D., Oh J.Y., Jeon E.J., Ryu H.S., Lee D.W., Kim B.S., Lee C.H. (2017). Comparative evaluation of microbial diversity and metabolite profiles in *doenjang*, a fermented soybean paste, during the two different industrial manufacturing processes. Food Chem..

[24] Razei A., Sorouri R., Mousavi S.L., Nazarian S., Amani J., Aghamollaei H. (2017). Presenting a rapid method for detection of *Bacillus cereus*, *Listeria monocytogenes* and *Campylobacter jejuni* in food samples. Iran. J. Basic Med. Sci..

[25] Ramarao N., Vincent S. (2013). The pore-forming haemolysins of *Bacillus cereus*: A Review. Toxins.

[26] Korea Food and Drug Administration (KFDA) (2010). Foodborne Pathogen Test Methods, Seoul, Republic of Korea. http://www.kfda.go.kr.

[27] Bahuguna A., Joe A.-R., Kumar V., Lee J.S., Kim S.-Y., Moon J.-Y., Cho S.-K., Cho H., Kim M. (2020). Study on the identification methods for effective microorganisms in commercially available organic agriculture materials. Microorganisms.

[28] Park K.M., Kim H.J., Jeong M.C., Koo M. (2016). Occurrence of toxigenic *Bacillus cereus* and *Bacillus thuringiensis* in *Doenjang*, a Korean fermented soybean paste. J. Food Prot..

[29] Kim M., Kim Y.S. (2012). Detection of foodborne pathogens and analysis of aflatoxin levels in home-made *Doenjang* samples. Prev. Nutr. Food Sci..

[30] Beaud D., Tailliez P., Anba-Mondoloni J. (2005). Genetic characterization of the β-glucuronidase enzyme from a human intestinal bacterium, *Ruminococcus gnavus*. Microbiology.

[31] Degrassi G., Uotila L., Klima R., Venturi V. (1999). Purification and properties of an esterase from the yeast *Saccharomyces cerevisiae* and identification of the encoding gene. Appl. Environ. Microbiol..

[32] Menteşe E., Baltaş N., Emirik M. (2020). Synthesis, α-glucosidase inhibition and in silico studies of some 4-(5-fluoro-2-substituted-1*H*-benzimidazol-6-yl) morpholine derivatives. Bioorg. Chem..

[33] Son S.H., Jeon H.L., Yang S.J., Lee N.K., Paik H.D. (2017). In vitro characterization of *Lactobacillus brevis* KU15006, an isolate from kimchi, reveals anti-adhesion activity against foodborne pathogens and antidiabetic properties. Microb. Pathog..

[34] Devanthi P.V.P., Gkatzionis K. (2019). Soy sauce fermentation: Microorganisms, aroma formation, and process modification. Food Res. Int..

[35] Alzeer J., Hadeed K.A. (2016). Ethanol and its Halal status in food industries. Trends Food Sci..

[36] Mayne S.T., Risch H.A., Dubrow R., Chow W.H., Gammon M.D., Vaughan T.L., Farrow D.C., Schoenberg J.B., Stanford J.L., Ahsan H. (2001). Nutrient intake and risk of subtypes of esophageal and gastric cancer. Cancer Epidemiol. Biomarkers Prev..

[37] Buratti S., Benedetti S., Méndez M.L.R. (2016). Alcoholic fermentation using electronic nose and electronic tongue. Electronic Noses and Tongues in Food Science.

[38] Hong Y., Jung H.-J., Kim H.-Y. (2012). Aroma characteristics of fermented Korean soybean paste (*Doenjang*) produced by *Bacillus amyloliquefaciens*. Food Sci. Biotechnol..

[39] Jo Y.-J., Cho I.H., Song C.K., Shin H.W., Kim Y.-S. (2011). Comparison of fermented soybean paste (*Doenjang*) prepared by different methods based on profiling of volatile compounds. J. Food Sci..

[40] Mah J.-H., Park Y.K., Jin Y.H., Lee J.-H., Hwang H.-J. (2019). Bacterial production and control of biogenic amines in Asian fermented soybean foods. Foods.

[41] Bahuguna A., Shukla S., Lee J.S., Bajpai V.K., Kim S.Y., Huh Y.S., Han Y.K., Kim M. (2019). Garlic augments the functional and nutritional behavior of *Doenjang*, a traditional Korean fermented soybean paste. Sci. Rep..

[42] Santos M.S. (1996). Biogenic amines: Their importance in foods. Int. J. Food Microbiol..

[43] Cho T.-Y., Han G.-H., Bahn K.-N., Son Y.-W., Jang M.-R., Lee C.-H., Kim S.-H., Kim D.-B., Kim S.-B. (2006). Evaluation of biogenic amines in Korean commercial fermented foods. Korean J. Food Sci. Technol..

[44] Kim T.-K., Lee J.-I., Kim J.-H., Mah J.-H., Hwang H.-J., Kim Y.-W. (2011). Comparison of ELISA and HPLC methods for the determination of biogenic amines in commercial *doenjang* and *gochujang*. Food Sci. Biotechnol..

[45] Lee H.T., Kim J.H., Lee S.S. (2009). Analysis of microbiological contamination and biogenic amines content in traditional and commercial *Doenjang*. J. Food Hyg. Saf..

[46] Food and Agriculture Organization of the United Nations/World Health Organization (FAO/WHO) (2013). Joint FAO/WHO Expert Meeting on the Public Health Risk of Histamine and Other Biogenic Amines from Fish and Fishery Products.

[47] Lee J.I., Oh Y.-K., Kim J.-H., Kim Y.-W. (2013). Rapid enzymatic assay of biogenic amines in *doenjang* and *gochujang* using amine oxidase. Food Sci. Biotechnol..

[48] Del Rio B., Redruello B., Fernandez M., Martin M.C., Ladero V., Alvarez M.A. (2020). The biogenic amine tryptamine, unlike β-phenylethylamine, shows in vitro cytotoxicity at concentrations that have been found in foods. Food Chem..

[49] Ramalingam S., Bahuguna A., Lim S., Joe A.R., Lee J.S., Kim S.Y., Kim M. (2021). Quantification of biogenic amines in 35 Korean cottage industry traditional gochujang (fermented red pepper paste) products. Foods.

[50] Jung W.Y., Jung J.Y., Lee H.J., Jeon C.O. (2016). Functional characterization of bacterial communities responsible for fermentation of *doenjang*: A traditional Korean fermented soybean paste. Front. Microbiol..

[51] Su-Yeon K., Hyeong-Eun K., Yong-Suk K. (2017). The potentials of *Bacillus licheniformis* strains for inhibition of *B. cereus* growth and reduction of biogenic amines in cheonggukjang (Korean fermented unsalted soybean paste). Food Control.

