# Supplementary material for: Safety Analysis of Korean Cottage Industries’ Doenjang, a Traditional Fermented Soybean Product: A Special Reference to Biogenic Amines
Source: Foods. 2023 Nov 10;12(22):4084. doi: 10.3390/foods12224084 (PMC10670832; doi:10.3390/foods12224084)
Supplement: Supplementary file 1 [file foods-12-04084-s001.zip › foods-2675394-supplementary.pdf]

Supplementary Data

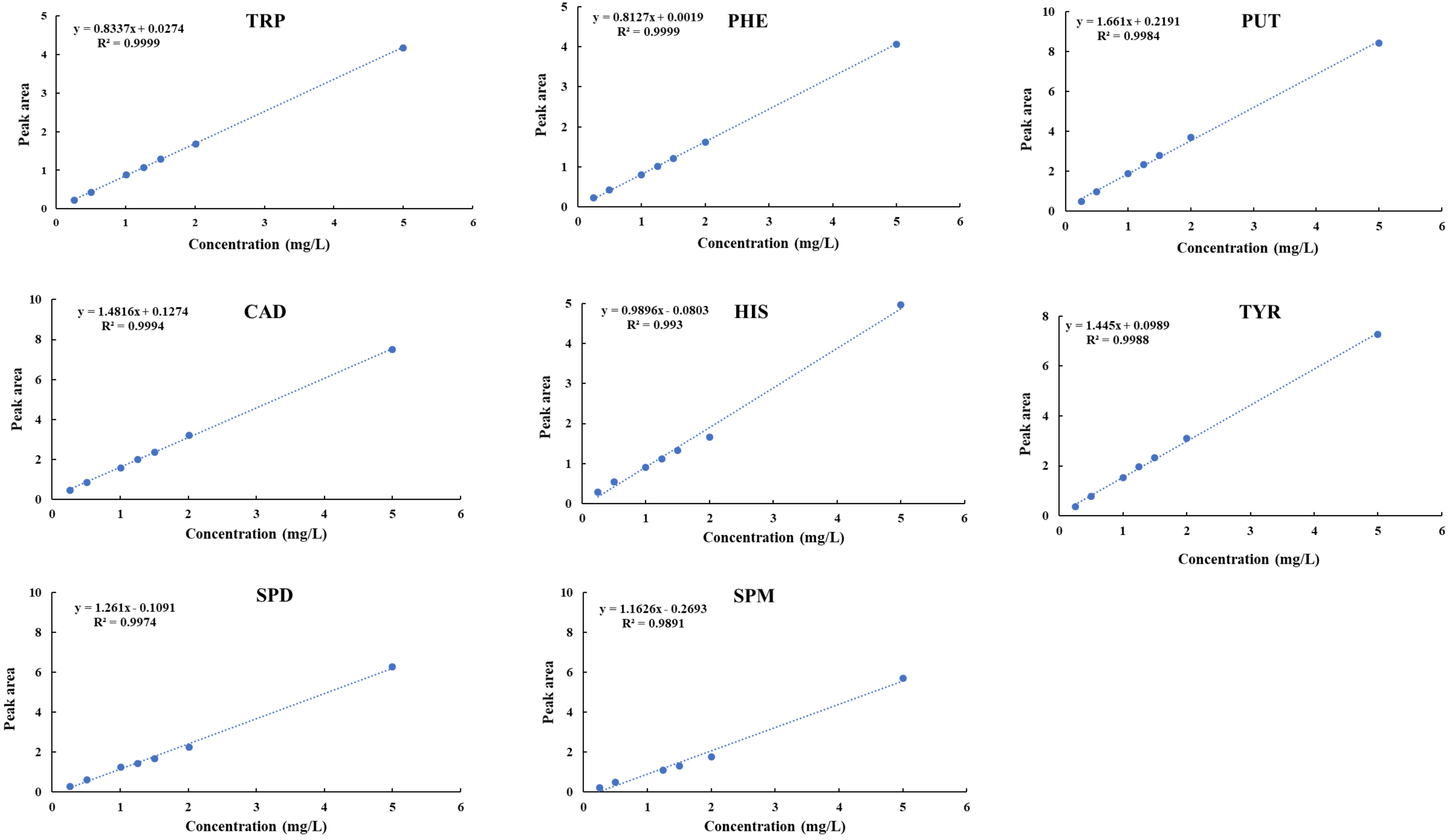

Supplementary Figure S1: Standard curves of different biogenic amines

Supplementary Table S1: Details of the selected *doenjang* samples.

Sample code	Location of the company (in Republic of Korea)	Ingredients
De-1	*Sancheong-gun, Gyeongsangnam-do*	Soybean, salt
De-2	*Buyeo-gun, Chungcheongnam-do*	Soybean, sea salt, purified water
De-3	*Jeongeup-si, Jeollabuk-do*	Soybean, sea salt, purified water
De-4	*Gyeongju, Gyeongsangbuk-do*	Soybean, sea salt
De-5	*Sancheong-gun, Gyeongsangnam-do*	Soybean, salt
De-6	*Goryeong-gun, Gyeongsangbuk-do*	Soybean, refined salt, purified water
De-7	*Gwangju*	Soybean, sea salt, purified water, mulberry leaf extract
De-8	*Yangpyeong-gun, Gyeonggi-do*	Soybean, sea salt, purified water
De-9	*Naju, Jeollanam-do*	Soybean, *meju*, purified water, sea salt
De-10	*Jeju, Jeju-do*	Soybean, refined salt
De-11	*Cheongdo-gun, Gyeongsangbuk-do*	Soybean, salt, purified water
De-12	*Hongcheon-gun, Gangwon-do*	Soybean, sea salt, purified water
De-13	*Cheongdo-gun, Gyeongsangbuk-do*	Soybean, sea salt, purified water
De-14	*Goryeong-gun, Gyeongsangbuk-do*	*Meju*, refined salt
De-15	*Gangjin-gun, Jeollanam-do*	Soybean, salt
De-16	*Chilgok-gun, Gyeongsangbuk-do*	Soybean, salt
De-17	*Gimcheon-si, Gyeongsangbuk-do*	Soybean, sea salt, purified water
De-18	*Chilgok-gun, Gyeongsangbuk-do*	Soybean, sea salt, purified water
De-19	*Yeongju, Gyeongsangbuk-do*	*Meju*, sea salt, purified water
De-20	*Andong, Gyeongsangbuk-do*	Soybean, sea salt, purified water
De-21	*Geumsan-gun, Chungcheongnam-do*	Soybean, sea salt, red chilli seed powder
De-22	*Jincheon-gun, Chungcheongbuk-do*	Soybean, traditional soy sauce
De-23	*Mungyeong-si, Gyeongsangbuk-do*	Soybean, sea salt
De-24	*Sunchang-gun, Jeollabuk-do*	Soybean, sea salt, purified water
De-25	*Sunchang-gun, Jeollabuk-do*	Soybean, sea salt, purified water

De-26	*Sunchang-gun, Jeollabuk-do*	Soybean, sea salt, purified water
De-27	*Sunchang-gun, Jeollabuk-do*	Soybean, sea salt, purified water
De-28	*Yongin-si, Gyeonggi-do*	*Meju*, salt, water, red chilli, jujube, roasted sesame seeds
De-29	*Jeju, Jeju-do*	Soybean, salt
De-30	*Sunchang-gun, Jeollabuk-do*	Soybean, refined salt, purified water
De-31	*Wonju-si, Gangwon-do*	*Meju*, purified water, sea salt
De-32	*Hapcheon-gun, Gyeongsangnam-do*	*Meju*, salt
De-33	*Sacheon, Gyeongsangnam-do*	Soybean, sea salt, water
De-34	*Sunchang-gun, Jeollabuk-do*	Soybean, sea salt
De-35	*Taean-gun, Chungcheongnam-do*	Soybean, sea salt
De-36	*Iksan-si, Jeollabuk-do*	Soybean, sea salt

Supplementary Table S2. pH, salinity, color values and amino nitrogen content of *doenjang* samples.

Sample name	pH values	Salinity (%)	L* values	Color		Amino nitrogen (mg/100g)
				a* values	b* values	
De-1	6.29±0.01^{c}	14.63±0.54$^{fgh)}$	35.57±0.26^{j}	8.03±0.05^{hi}	11.51±0.10^{g}	261.86±16.20^{mno}
De-2	5.23±0.00^{n}	15.89±0.28^{c}	29.72±0.15^{v}	4.24±0.02^{o}	5.82±0.04^{qrs}	621.91±37.11^{bc}
De-3	4.65±0.01^{z}	13.93±0.25^{hij}	30.35±0.23^{u}	7.67±0.19^{o}	8.37±0.27^{lm}	210.42±8.10^{oC}
De-4	5.10±0.01^{q}	15.10±0.67^{def}	34.65±0.42^{l}	6.36±0.14^{p}	8.56±0.14^{tu}	289.91±8.10^{lmn}
De-5	5.52±0.01^{l}	12.82±0.31^{klm}	34.81±0.18^{kl}	5.51±0.02^{b}	9.37±0.18^{a}	346.02±29.20^{ijkl}
De-6	6.21±0.02^{e}	14.76±0.55^{efg}	33.96±0.14^{mno}	5.52±0.15^{bc}	8.86±0.09^{b}	420.84±8.10^{gh}
De-7	5.44±0.00^{m}	13.17±0.29^{jkl}	33.73±0.03^{nop}	5.95±0.07^{ij}	8.68±0.03^{mn}	663.99±24.30^{b}
De-8	4.83±0.01^{w}	15.76±0.28^{cd}	31.26±0.27^{s}	4.85±0.11^{no}	6.91±0.06st	495.66±37.11^{ef}
De-9	5.04±0.01^{r}	12.55±0.78^{lmn}	32.00±0.15^{r}	3.95±0.15^{s}	4.83±0.22^{y}	476.95±35.30^{fg}
De-10	4.97±0.01^{u}	15.43±0.25^{cde}	33.60±0.36^{op}	6.24±0.04^{lm}	8.64±0.27^{hi}	243.15±24.30^{gh}
De-11	6.31±0.01^{b}	13.27±0.28^{jkl}	37.87±0.19^{g}	6.22±0.04^{fg}	9.77±0.06^{d}	420.84±69.20^{gh}
De-12	4.79±0.00^{y}	11.97±0.33^{no}	33.20±0.23^{q}	5.84±0.69^{t}	8.24±0.67^{x}	420.84±8.10^{fg}
De-13	5.16±0.01^{p}	16.03±0.00^{bc}	45.43±0.32^{b}	9.48±0.22^{h}	17.26±0.08^{op}	289.91±49.26^{lmn}
De-14	5.11±0.00^{q}	14.21±0.26^{ghi}	46.32±0.21^{a}	9.32±0.12^{n}	16.89±0.14st	336.67±53.11^{ijkl}
De-15	4.99±0.01^{t}	14.99±0.63^{efg}	34.00±0.32^{mno}	7.81±0.28^{g}	9.48±0.26^{c}	341.35±42.08^{ijkl}
De-16	4.96±0.00^{u}	14.26±0.31^{gh}	34.16±0.18^{m}	6.48±0.06^{ef}	8.53±0.08^{jk}	289.91±32.40^{lmn}
De-17	5.78±0.02^{h}	13.50±0.50^{ijk}	29.78±0.32^{v}	4.50±0.13^{k}	5.48±0.15^{f}	392.78±16.20^{hi}
De-18	5.88±0.01^{g}	13.44±0.26^{jk}	36.10±0.09^{i}	7.09±0.06^{ij}	10.65±0.16^{k}	743.48±21.43^{aC}
De-19	5.90±0.01^{f}	12.63±0.64^{lmn}	41.32±0.31^{d}	8.59±0.06^{no}	13.75±0.32^{v}	439.54±24.30^{gh}
De-20	4.79±0.01^{y}	12.72±0.76^{klmn}	33.38±0.10^{pq}	8.19±0.13^{cd}	9.16±0.10^{e}	355.38±56.11^{ijk}
De-21	5.67±0.01^{i}	13.29±0.50^{jkl}	35.05±0.33^{k}	6.55±0.11^{kl}	8.54±0.14^{mn}	495.66±28.06^{ef}
De-22	5.26±0.01^{v}	15.93±0.52^{c}	34.79±0.31^{kl}	7.84±0.02^{rA}	10.17±0.17^{x}	308.62±21.43^{klm}
De-23	4.96±0.01^{u}	11.56±0.30^{op}	32.15±0.11^{r}	6.34±0.05^{g}	7.77±0.09^{hij}	584.5±42.86^{cd}

De-24	4.97±0.00^{u}	12.54±0.00^{lmn}	38.64±0.28^{f}	9.07±0.07^{a}	12.77±0.07^{ijk}	252.5±35.30^{mno}
De-25	6.24±0.01^{d}	11.14±0.24^{pq}	34.10±0.01^{mn}	7.33±0.02^{m}	9.48±0.05^{pq}	617.23±49.26^{bc}
De-26	5.88±0.01^{g}	10.83±0.00^{q}	38.87±0.31^{f}	6.27±0.02^{deB}	10.77±0.03^{f}	341.35±24.30^{ijkl}
De-27	5.11±0.01^{q}	12.95±0.27^{klm}	32.09±0.11^{r}	6.97±0.02^{n}	8.17±0.02^{g}	528.39±53.11^{def}
De-28	5.54±0.01^{k}	13.00±0.46^{klm}	30.86±0.02^{t}	4.88±0.02^{v}	5.98±0.02^{a}	565.8±14.03^{fg}
De-29	4.81±0.01^{x}	16.65±0.43^{ab}	33.89±0.20^{mno}	8.47±0.25^{defA}	10.59±0.17^{d}	598.53±35.30^{ef}
De-30	5.18±0.01^{o}	11.73±0.29^{op}	33.11±0.08^{q}	9.75±0.02^{o}	10.43±0.01^{l}	224.45±29.20^{o}
De-31	5.01±0.00^{s}	14.45±0.30^{fgh}	36.55±0.12^{h}	7.07±0.05^{no}	8.94±0.08^{rst}	322.64±29.20^{jkl}
De-32	5.87±0.01^{g}	12.26±0.26^{mno}	37.99±0.17^{g}	8.87±0.06^{q}	12.09±0.07^{no}	542.42±21.43^{de}
De-33	5.57±0.01^{j}	13.90±0.29^{hij}	38.78±0.04^{f}	6.53±0.03^{q}	11.72±0.02^{pqr}	416.16±8.10^{gh}
De-34	6.83±0.02^{a}	14.93±0.53^{efg}	29.74±0.16^{v}	3.63±0.03^{p}	4.12±0.01^{qrs}	579.82±14.03^{cd}
De-35	5.23±0.00^{n}	16.79±0.54^{a}	41.44±0.04^{d}	8.83±0.02^{r}	13.69±0.04^{w}	233.8±35.30^{no}
De-36	5.10±0.01^{g}	13.00±0.27^{klm}	38.64±0.18^{f}	6.24±0.10^{u}	9.82±0.17^{z}	383.43±37.11^{hij}

Different superscripts in the column represent the statically difference between the samples at p<0.05

Supplementary Table S3. Enzymes and their substrate

Sr. No.	Substrate	Enzyme
1.	2-Naphthyl phosphate	Alkaline phosphatase
2.	2-Naphthyl butyrate	Esterase
3.	2-Naphthyl caprylate	Esterase lipase
4.	2-Naphthyl myristate	Lipase
5.	L-Leucsyl-2-naphthylamide	Leucine arylamidase
6.	L-Valyl-2-naphthylamide	Valine arylamidase
7.	L-Cystyl-2-naphthylamide	Cystine arylamidase
8.	*N*-Benzoyl-DL-arginine-2-naphthylamide	Trypsin
9.	*N*-Glutaryl-phenylanine-2-naphthylamide	α-Chymotrypsin
10.	2-Naphthyl phosphate	Acid phosphatase
11.	Naphthol-AS-BI-phosphate	Naphthol-AS-BI-phosphohydrolase
12.	6-Br-2-Naphthyl-αD-galactopyranoside	α-Galactosidase
13	2-Naphthyl-βD-galactopyranoside	β-Galactosidase
14.	Naphthol-AS-BI-βD-glucuronide	β-Glucuronidase
15.	2-Naphthyl-αD-glucopyranoside	α-Glucosidase
16.	6-Br-2-Naphthyl-βD-glucopyranoside	β-Glucosidase
17.	1-Naphthyl-N-acetyl-βD-glucosaminide	*N*-Acetyl-β-glucosaminidase
18.	6-Br-2-naphthyl- αD-mannopyranoside	α-Mannosidase
19.	2-Naphthyl- αL-fucopyranoside	α-Fucosidase

Supplementary Table S4: Limit of detection (LOD) and limit of quantification (LOQ) for different biogenic amines

Biogenic amine	R^2	Standard error intercept	Standard deviation intercept	Slope	LOD (mg/kg)	LOQ (mg/kg)
**TRP**	0.9999	0.0096	0.0255	0.8337	0.1009	0.3059
**PHE**	0.9999	0.0086	0.0229	0.8127	0.0931	0.2823
**PUT**	0.9984	0.0650	0.1721	1.6610	0.3419	1.0362
**CAD**	0.9994	0.0351	0.0930	1.4816	0.2071	0.6277
**HIS**	0.9930	0.0819	0.2167	0.9896	0.7226	2.1898
**TYR**	0.9988	0.0498	0.1320	1.4450	0.3014	0.9135
**SPD**	0.9974	0.0641	0.1696	1.2610	0.4438	1.3449
**SPM**	0.9891	0.1436	0.3518	1.1626	0.9986	3.0261

Supplementary Methods

M 1. Determination of pH and salinity

The *doenjang* samples pH values were determined by using a pH meter (Thermo Electron Corporation, Beverly, MA, USA) (Lee et al., 2018).

The salinity of the *doenjang* samples was determined by the Korea Food and Drug Administration method (Korea Food and Drug Administration, 2019). In brief, 1 g of *doenjang* sample was used to prepare ash at 550°C, which was diluted to 500 fold with distilled water. Approximately 2-3 drops of potassium chromate solution were added to 10 mL of diluted ash sample and were titrated against 0.02 N silver nitrate, till the yellow color reaction mixture turned to brown color. The total percentage of salinity was calculated by using the following formula,

$$\text{Salinity (\%)} = (V \times F \times D \times 0.00117/S) \times 100$$

where, V: volume of 0.02 N $AgNO_3$, F: factor of 0.02 N $AgNO_3$ (at 25 °C). D: dilution factor, S: total weight of the sample.

M 2. Evaluation of the color value

The color values of *doenjang* were analyzed in a Konica Minolta Chroma Meter (Konica Minolta Inc., Tokyo, Japan). The tristimulus color analyzer was calibrated to a reference (white porcelain plate) before the experiment. The color coordinates were recorded with Hunter scale values (L*, a*, and b* values). The L* value designates the brightness that extended from black (L* = 0) to white (L* = 100); a* value specifies redness from green (a* = −60) to red (a* = +60); b* value indicates yellowness from b* = −60 (blue) to b* = +60 (yellow) (Lee et al., 2018).

M 3. Determination of free amino nitrogen

Total free amino nitrogen of *doenjang* samples was determined by the titration method described by Korea Food and Drug Administration (2014). About 4 g of *doenjang* sample was mixed and stirred with 200 mL of distilled water for 30 min and filtered, the pH of the filtered *doenjang* solution (50 mL) was adjusted to 8.5. The 35% formalin solution (20 mL) was added with the filtered *doenjang* solution and then titrated against NaOH (0.1 N) until a pH of 8.5 was attained. Total amino nitrogen content of the *doenjang* was determined by applying the following formula,

$$\text{Amino nitrogen (mg/100g)} = ((V_1 - V_2) \times F \times D \times 1.4)/S) \times 100$$

where, V_1: the volume of 0.1 N NaOH (without sample), V_2: the volume of 0.1 N NaOH (with sample), F: factor of 0.1 N NaOH, D: dilution factor, 1.4: nitrogen equivalent in 1 mL of 0.1 N NaOH, S: sample weight.

M 4. Crystalline protein staining

For the identification of *B. thuringiensis,* crystal protein staining was performed using USFDA (FDA, 2012) method as adopted by Bahuguna et al. (2020). Briefly, bacterial isolate was grown on nutrient agar medium and incubated at 30 °C for 24 h, followed by 5 days of incubation at room temperature. After incubation, bacterial smear was prepared, air-dried, and fixed by gentle heating. The bacterial smear was flooded with methanol for 30 s, followed by the addition of 0.5% basic fuchsin. Sides were heated gently until steam appeared. Finally, slides were rinsed with water and visualized under the microscope (SOMETECH, Seoul, Republic of Korea) for the presence of tetragonal (diamond-shaped) toxin crystals.
